# Hyperuricemia increases susceptibility to chronic kidney injury exacerbation via autophagic flux blockade-mediated ammonia death pathway

**DOI:** 10.1016/j.jare.2025.11.003

**Published:** 2025-11-07

**Authors:** Yu-xin Zhang, Hui Wan, Guan-yue Shan, Zhi-cheng Gao, Hai-jun Li

**Affiliations:** a Laboratory for Tumor Immunology, The First Hospital of Jilin University, Changchun 130021, China; b Institute of Ginseng Research, Jilin University, Changchun 130021, China

**Keywords:** Hyperuricemic nephropathy, Ammonia metabolism, Mitochondrial damage, Lysosomal alkalinization, Autophagic flux

## Abstract

*Introduction:* Ammonia (NH3), a core toxic byproduct of amino acid metabolism in the body, poses a severe threat to cell survival when its homeostasis is disrupted. Maintaining low systemic ammonia concentrations is crucial. Under physiological conditions, the kidneys regulate ammonia metabolism precisely through glutaminase (GLS1)- mediated ammonia production and the urea cycle, ensuring efficient detoxification. Hyperuricemic nephropathy (HN), a common complication of hyperuricemia, impairs patient health significantly. However, whether and how ammonia toxicity triggers cell death under this pathological condition remains unclear.

*Objectives:* This study aimed to investigate whether ammonia-dependent cell death occurs in HN and to elucidate the underlying molecular mechanisms, focusing on the role of ammonia metabolism disorder and autophagic flux blockade.

*Methods:* We employed both in vivo and in vivo models of HN. Cellular and tissue damage were assessed by ultrastructural analysis, fluorescent probes for reactive oxygen species and mitochondrial membrane potential, and autophagic flux detection. Causal relationships were verified using ammonia scavengers and gene intervention.

*Results:* Chronic hyperuricemic stress accelerated renal glutaminolysis to maintain ATP supply, leading to excessive mitochondrial ammonia production. Accumulated ammonia underwent RHCG-dependent transmembrane transport, causing lysosomal alkalinization and dysfunction, which in turn promoted mitochondrial ammonia retention and swelling. This process inhibited autolysosomal disassembly, impaired clearance of damaged mitochondria, and blocked autophagic flux, ultimately driving cell death.

*Conclusion:* Our findings identified a distinct form of cell death in HN, mechanistically divergent from previously known mechanisms such as apoptosis or pyroptosis. It redefined HN pathogenesis through a metabolic lens, identifying druggable targets to mitigate renal damage in hyperuricemic patients.

## Highlights

• Ammonia overload triggers lysosomal alkalinization in hyperuricemic nephropathy.
• HN accelerates renal glutamine catabolism, generating excess ammonia.
• Mitochondrial ammonia retention impairs autophagic flux and mitochondrial clearance.
• Ammonia induced cell death mechanism was found in HN.

## Graphical abstract

Schematic diagram illustrating how hyperuricemia exacerbates chronic kidney injury through an autophagy flux blockade-mediated ammonia-induced metabolic death pathway. MSU crystals and a hyperuricemic diet induce renal glutaminolysis disorder, leading to mitochondrial ammonia overload and damage. RHCG protein-mediated ammonia translocation into lysosomes causes lysosomal alkalinization and hydrolase inactivation. This results in accumulation of undigested autophagosomes and autolysosomes, impairing clearance of damaged mitochondria. Ultimately, metabolic waste accumulation and ATP depletion drive cell death.

**Figure:**
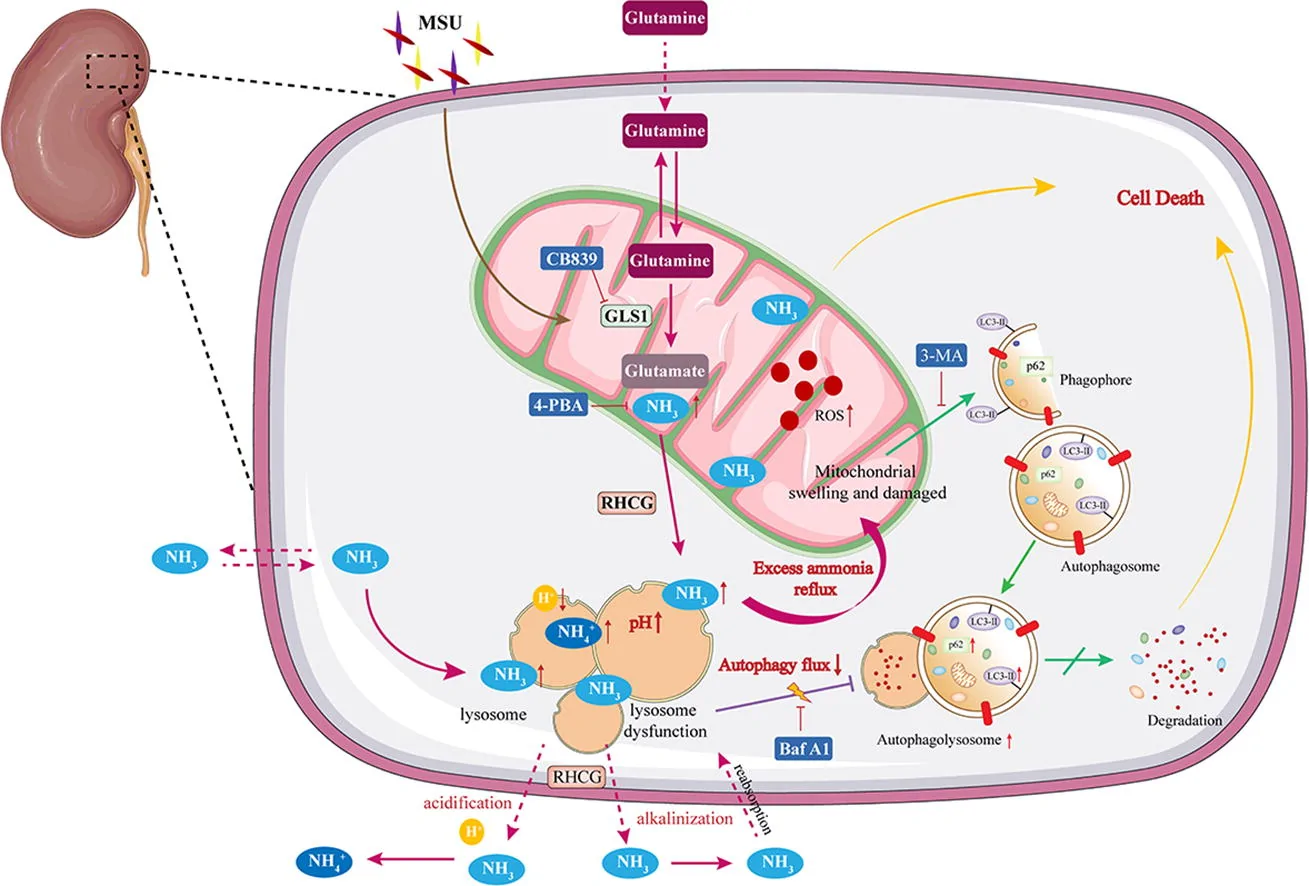

## Introduction

Hyperuricemia (HUA), a metabolic disorder characterized by elevated serum uric acid (UA) levels, exhibits increasing global prevalence and is strongly associated with chronic kidney disease (CKD) development [1,2]. Exceeding the solubility threshold of UA levels triggers the crystallization process, resulting in MSU crystal formation and their subsequent deposition in various tissues such as joints, soft tissues, and kidneys. Due to their critical role in both UA excretion and reabsorption processes, renal tissues exhibit particular susceptibility to UA accumulation. This deposition leads to renal injury, and chronic inflammation from persistent UA accumulation can further exacerbate CKD progression, eventually resulting in renal failure [3]. Understanding the pathogenesis of hyperuricemic nephropathy (HN) and identifying effective therapeutic strategies are crucial. Research indicates that HN exacerbates renal injury through multiple mechanisms, including oxidative stress, inflammatory responses, fibrosis, and the activation of various proteins and signaling pathways [4,5]. Specifically, UA crystals in the kidney can activate the NLRP3 inflammasome, triggering the release of pro-inflammatory cytokines such as IL-1β and TNF-α, which promote tubulointerstitial fibrosis, apoptosis, and pyroptosis [6]. Additionally, HUA disrupts renal energy metabolism, inducing cell death via mitochondrial dysfunction, reactive oxygen species (ROS) generation, and endoplasmic reticulum stress [7,8].

Ammonia (NH_3_), a key by-product of amino acid metabolism, plays a critical role in maintaining cellular homeostasis. Under normal physiological conditions, the kidney regulates systemic ammonia metabolism through glutaminase-mediated ammonia production and the urea cycle [9]. However, in the case of hyperuricemic renal injury, when the balance of renal ammonia metabolism is broken, there will be disorder and damage of ammonia metabolism, but the specific regulation imbalance and the cell death mode have not been fully clarified. In terms of high ammonia alone, a large number of studies are currently focused on its ability to damage astrocytes and other neural cells, leading to hepatic encephalopathy [10,11], or high ammonia injury of cells in the liver [12,13]. Generally speaking, the cells of other tissues are considered to decompose amino acids to generate glutamine, which is transported to the liver through the blood circulation and then releases ammonia for the urea cycle [14]. Some extrahepatic cells can also produce ammonia through glutaminolysis, but the ammonia produced is difficult to have a good way to achieve complete clearance [15,16]. Unlike other tissues, the kidney can finely regulate ammonia metabolism to maintain systemic acid-base balance. In proximal tubular cells, glutaminase 1 (GLS1) catalyzes the conversion of glutamine to glutamate, releasing ammonium ions (NH_4_^+^), which are subsequently converted into α-ketoglutarate by glutamate dehydrogenase (GDH) and enter the tricarboxylic acid cycle [[17], [18], [19]]. Upon kidney injury, this metabolic process becomes dysregulated, resulting in ammonia accumulation and exacerbating tissue damage, although the underlying mechanisms remain poorly understood.

To investigate whether ammonia metabolism is disrupted in hyperuricemic kidney injury and to elucidate the mechanisms of ammonia-induced cell damage, we conducted in vivo and in vitro analyses using HK-2 cells and kidney tissues exposed to MSU crystals and a high UA diet. Our findings revealed that glutamine, within the mitochondria, was catabolized by GLS1 to fuel the tricarboxylic acid cycle, generating ammonia as a by-product. This ammonia was subsequently transported to lysosomes via the RHCG (Rhesus Monkey Family Glycoprotein) transporter to maintain acid-base balance. In the context of HUA, enhanced glutaminolysis increases ammonia production, which accumulates within the lysosomes. This accumulation leads to lysosomal alkalinization, reduced membrane permeability, and impaired function, further exacerbating cellular damage. Additionally, the excessive ammonia load overwhelms the kidney’s ability to clear it, causing mitochondrial swelling and dysfunction. Normally, damaged mitochondria are degraded through autophagy; however, our study indicated that mitophagy was impaired under these conditions. The accumulation of dysfunctional mitochondria contributed to further renal injury, exacerbating the progression of HN.

## Materials and methods

A comprehensive diagrammatic summary of the experimental design is illustrated in Fig. S1.

### Reagents and antibodies

RHCG Polyclonal antibody were purchased from proteintech. LC3A/B (microtubule-associated protein 1 light chain 3, D3U4C) XP® Rabbit mAb were purchased from Cell Signaling Technology. GAPDH antibody (TA802519) were purchased from ORIGENE. V-ATPase B2 antibody (sc-166122) were purchased from Santa Cruz Biotechnologies. Glutaminase Rabbit mAb (R381275), SQSTM1/p62 Rabbit pAb (380612) were purchased from Zenbioscience. 4-PBA (HY-A0281), CB-839 (C872897), 3-Methyladenine (3-MA, HY-19312), Bafilomycin A1 (BafA1, HY-100558), acridine orange hydrochloride (AO, HY-101879) were purchased from MedChemExpress. LysoSensor™ Green DND-189 (40767ES50) were purchased from YEASEN. Peroxidase-conjugated goat anti-rabbit immunoglobulin G (IgG) (H + L) (AP31L117) and anti-mouse IgG (H + L) (AP31L119) were purchased from Life-iLab. CCK-8 test kit (C0037), ROS test kit (S0033S), JC-1 staining test kit (C2006), yeast extract (YE, ST968), Mito-Tracker Red CMXRos(C1035-50ug), Lyso-Tracker Red(C1046), PI staining test kit (ST511) and Hoechst 33,258 staining test kit (C1011) were purchased from Beyotime Biotechnology. Adenine (Ad, A800684) were purchased from Mackin. The assays for measuring Uric acid Test Kit (UA, C012-2–1), Creatinine Assay kit (CRE, C011-2–1), Xanthine Oxidase assay kit (XOD, A002-1–1) and Urea Assay Kit (BUN, C013-2–1) were conducted using kits obtained from Nanjing Jiancheng Bioengineering Institute.

### Cell culture and processing

Human proximal tubule epithelial cell line (HK-2) cells were cultured in a humidified environment at 37°C and 5 % CO_2_. The cells were purchased from Pricella (CL-0109). The culture medium consisted of Dulbecco's Modified Eagle Medium (DMEM) supplemented with 10 % foetal bovine serum (FBS, Pricella, USA). Prior to other treatments, cells were starved for 24 h using medium containing 0.5 % FBS.

HK-2 cells were treated with monosodium urate (MSU, HY-B2130A, MCE) at concentrations of 0.05, 0.1, 0.2 mg/ml for 24 h to induce hyperuricemic renal injury changes [20,21]. To assess the cytotoxic effects of MSU, cells were treated with different concentrations of MSU (0, 0.05, 0.1, 0.2, 0.4, 0.8, 1.0 mg/ml) for 24 h. To evaluate the toxic effects of MSU on HK-2 cells at different time points, cells were treated with 0.2 mg/ml MSU for 0, 6, 12, 24, 48, and 96 h. To elucidate the mechanisms underlying MSU crystal- and high-purine diet-induced cell death, we selected 2 μM 4-PBA and 2 μM CB-839 for pretreatment [24,25]. To explore the occurrence period of autophagy blockage, we selected 5 mM 3-MA and 10 nM Baf A1 for pretreatment [26,27].

### Cell viability detection

HK-2 cells were seeded onto a 96-well plate and allowed to adhere. Subsequently, the cells were divided into groups and subjected to drug intervention as per the experimental design. Cell viability was assessed using the CCK-8 assay kit.

### RHCG overexpression in HK-2

HK-2 cells were inoculated into a 6-well plate (8 × 10^5^ cells per well), then transfect with 50 ng of plasmid using the Lipofectamine 2000 kit (Thermo Scientific, USA) for 6 h according to the manufacturer's guidelines. pcDNA3.1(+)-human RHCG was purchased from IBSBIO (China). The culture medium was replaced with medium containing or without MSU crystals (0.2 μM) for 48 h. After treatment, the cells were washed twice and then used for subsequent procedures.

### Animal studies

Male C57BL/6J mice (6–8 weeks old) were purchased from Jilin Qianhe Model Biotechnology Co., Ltd. (Changchun, China), quarantined, and acclimated for 1 week. All animal experiments were approved by the Animal Care and Use Committee of the First Hospital of Jilin University (Approval No. 11569). The mice were housed at 20-23°C and 40–80% humidity with free access to food and water. To establish a HN model, we administered a high-purine diet according to a predefined protocol [6,22,23]. C57BL/6J mice were randomly divided into eight groups (n = 5): Control (normal saline, i.g.), YE/A low-dose (YE 10 g/kg + ad 50 mg/kg, i.g.), YE/A middle-dose (YE 15 g/kg + ad 75 mg/kg, i.g.), YE/A high-dose (YE 20 g/kg + ad 100 mg/kg, i.g.), 4-Phenylbutyric acid (4-PBA) (YE 20 g/kg, i.g. + ad 100 mg/kg, i.g. + 4-PBA 200 mg/kg, i.p.) [24], CB-839 (YE 20 g/kg, i.g. + ad 100 mg/kg, i.g. + CB-839 200 mg/kg, i.p.) [25], 3-MA (YE 20 g/kg, i.g. + ad 100 mg/kg, i.g. + 3-MA 1 mg/kg, i.p.) [26], and Baf A1 (YE 20 g/kg, i.g. + ad 100 mg/kg, i.g. + Baf A1 30 mg/kg, i.p.) [27]. YE, adenine, 4-PBA, CB-839, 3-MA, and Baf A1 were dissolved in 0.9 % normal saline. 4-PBA, CB-839, 3-MA, and Baf A1 were administered via pre-injection 3 h prior to oral administration of YE and ad. The experimental procedure was repeated daily for 8 days. Body weight was recorded daily during the experimental period. On day 9, blood was collected from the eyes of all experimental mice, and kidney tissue was collected via decapitation.

### Reactive oxygen species (ROS), mitochondrial membrane potential (MMP) detection

According to the manufacturer's instructions, the fluorescent probe DCFH-DA measures ROS production, which is oxidised by intracellular ROS. Cells were seeded in 24-well plates and treated with MSU crystals. After treatment, cells were washed twice with PBS and incubated with 10 μM DCFH-DA in basal DMEM at 37°C in the dark for 30 min. Similarly, MMP was assessed using the JC-1 staining kit. Fluorescent cells were observed under a fluorescence microscope and quantified using Image J software.

### Measurement of biochemical indicators

Use specific test kits to assess the levels of UA, BUN, CRE, and XOD in serum and cell particles. Use the BCA method to quantify protein concentration in tissues and cells, and make adjustments before the experiment. Add the test reagent to the supernatant according to the kit instructions, then incubate for an appropriate period of time and measure the optical density (OD) value.

### Ammonia determination

The ammonia determination kit (Cohesion Biosciences, CAK1181) is used for quantitative ammonia measurement. In simple terms, cells are collected and lysed on ice, and then ammonia is detected using equal amounts (approximately 5 × 10⁶) of HK-2 cells from different groups. The ammonia content in whole-cell lysates or subcellular organelles is detected according to the method provided by the manufacturer.

### Cathepsin D activity assay

The activity of cathepsin D (Cath-D) was measured using a commercial ELISA kit (Nanjing Jiancheng Bioengineering Institute, H617-1–1) according to the manufacturer's instructions. Briefly, treated HK-2 cells were lysed and centrifuged. The supernatant was incubated with a specific enzyme reagent, and the reaction was quantified by measuring the optical density at 450 nm. Cath-D activity was calculated based on a standard curve. Experiments were performed with at three independent biological replicates.

### Isolation of organelles from HK-2 cells

#### Mitochondrial extraction

Use the Cell Mitochondria Isolation Kit (Beyotime, C3601) and follow the manufacturer's protocol for extraction. In brief, homogenise 5 × 10⁶ HK-2 cells using a homogeniser, lyse with lysis buffer, then centrifuge at 600 g and 4°C for 10 min to collect the supernatant. Centrifuge the supernatant at 11,000 g and 4°C for 10 min to collect the mitochondria.

#### Lysosome extraction

Use the lysosome extraction kit (Solarbio, EX2670) according to the manufacturer's protocol. In brief, wash the cells three times with PBS, then add 400 μL of cold reagent A and incubate at 4°C for 10 min. Next, homogenise the cell suspension 30–40 times using a homogeniser and centrifuge the homogenate at 1,000 g for 5 min at 4°C. Centrifuge the supernatant at 3,000 g and 5,000 g for 10 min, remove the pellet, and collect the supernatant. Subsequently, centrifuge the supernatant at 25,000 g for 20 min, collect the precipitate, and resuspend it in 400 μL of cold reagent B. Finally, centrifuge the supernatant at 25,000 g for 20 min at 4°C, collect the precipitate, and resuspend it in 100 μL of lysosome preservation solution C for subsequent experiments.

### Western blot (WB)

HK-2 cells or kidney tissue were lysed and dissolved in pre-chilled lysis buffer composed of RIPA and PMSF (100:1) to extract total protein for 30 min. The mixture was centrifuged at 12,000 rpm for 10 min, and the supernatant was collected. Protein concentration was detected using the BCA kit (Beyotime Biotechnology, P0012). The prepared proteins were denatured by boiling. Protein samples were separated by 12 % SDS-PAGE (Life-iLab, AP15L545) and transferred to a PVDF membrane (Cytiva, 10600023). The membrane was blocked with 5 % milk dissolved in PBST for 1 h. The primary antibody was added and incubated overnight at 4°C. After washing with PBST, the appropriate secondary antibody was incubated at room temperature for 1 h. Immunoblots were detected using an enhanced chemiluminescence detection kit (Life-iLab, AP34L025) with an imaging system. PageRuler™ pre-stained protein molecular weight standards (Thermo Scientific, 26617) were used as molecular weight standards. GAPDH was used as a loading control. Following the latest guidelines for the use and interpretation of autophagy monitoring assays (4th edition) [28], LC3-II expression normalised to housekeeping protein was used as an indicator of autophagy flux.

### Immunofluorescence staining (IF)

HK-2 cells were seeded into a 12-well plate and allow them to adhere overnight, then cultured according to the method described in 2.2. Fixed the cells in pre-chilled methanol for 10 min, then permeabilised them in 0.15 % Triton X-100 for 10 min. After incubating with a blocking solution containing 5 % bovine serum albumin (BSA) at room temperature for 30 min, the cells were incubated with specific primary antibodies (RHCG at 1:500, LC3 at 1:200) at 4°C overnight, followed by incubation with the corresponding secondary antibodies for 1 h. Lysosomes were identified using Lyso-Tracker Green, mitochondria using Mito-Tracker Red CMXRos, and cell nuclei using DAPI/Hoechst 33342. Fluorescent cell expression was observed using a fluorescence microscope.

### Immunohistochemistry (IHC)

In immunohistochemical analysis, paraffin sections were subjected to dewaxing and rehydration followed by antigen retrieval. Endogenous peroxidase was inactivated by treatment with a 3 % hydrogen peroxide solution in methanol for 15 min. Sections were blocked with 5 % bovine serum albumin (BSA) for 1 h, followed by overnight incubation at 4°C with anti-GLS1, RHCG, and LC3 A/B antibodies. After incubation with secondary antibodies, the sections were stained with DAB and haematoxylin to obtain images. The images were acquired using an AVIBENT microscope (Japan).

### Histopathological examination (HE)

The kidney tissue specimens were initially fixed in 4 % paraformaldehyde solution and subsequently embedded in paraffin to prepare tissue sections. Histological evaluation of liver morphology was conducted using hematoxylin and eosin (H&E) staining technique. Microscopic examination of the stained sections was performed under a light microscope.

### Transmission electron microscopy (TEM)

All cells or tissues treated with or without MSU crystals, a high-purine diet, and inhibitors were fixed overnight in 2.5 % glutaraldehyde, then embedded in resin and subjected to sequential dehydration and ultrathin sectioning (Leica, Leica EM UC7). The samples were then visualised using a transmission electron microscope (Hitachi, HT7800; Japan; magnification, 5,000×, 15,000× ).

### Lyso-Tracker Red, LysoSensor® GREEN, and AO staining

To observe the morphology of lysosomes, cells were stained with pre-warmed 50 nm Lyso-Tracker Red in 37°C cell culture medium for 30 min, followed by observation under a fluorescence microscope. To analyse lysosomal pH, cells were stained with 1 μM LysoSensor® GREEN in 37°C cell culture medium for 30 min, resuspended, and analysed by flow cytometry (FCM). To detect lysosomal membrane permeability, cells were stained with 5 μM AO in culture medium for 20 min and then observed under a fluorescence microscope.

### Statistical analysis

The data were analyzed utilizing GraphPad Prism version 9.5 software, and the results were presented as mean ± standard deviation (SD). Multiple group comparisons were performed using one-way analysis of variance (ANOVA). Each experiment was replicated three times to ensure statistical robustness. Statistical significance was denoted as follows: (*P < 0.05, ^**^P < 0.01, ^***^P < 0.001 vs. Control group; ^#^P < 0.05, ^##^P < 0.01, ^###^P < 0.001 vs. MSU group).

## Results

### MSU crystals and high-purine diet induce intracellular vacuolization, ammonia dysregulation, and cell death in vivo and in vitro

Numerous studies have demonstrated that elevated UA concentrations can lead to hyperuricaemic nephropathy, wherein UA exceeding its solubility threshold forms MSU crystals, contributing to kidney injury. MSU crystal deposition is a key factor in hyperuricemic kidney damage, with the stimulation of kidney cells by MSU crystals effectively replicating the hyperuricemic cellular environment in vitro. The chemical structure of MSU crystals is depicted in Fig. S2A. To simulate this hyperuricemic environment in vitro, we assessed the cytotoxicity of different MSU concentrations using the CCK8 assay on HK-2 cells over 24 h. We observed a stabilization of the cell death rate at 0.1 μM MSU, with a significant decline at 1 μM, allowing us to select 0.05, 0.1, and 0.2 μM as low, medium, and high concentrations for subsequent experiments (Fig. 1A). MSU crystal stimulation led to concentration-dependent ammonia accumulation and cell death in HK-2 cells (Fig. 1B–D), although ammonia levels in the culture medium did not increase (Fig. 1E). Considering the cytotoxicity of ammonia, we hypothesized that MSU-induced stimulation could raise intracellular ammonia levels, thereby contributing to cell death. Indeed, exogenous NH_4_Cl treatment induced cell death in HK-2 cells (Fig. 1F). Morphologically, we observed that dead HK-2 cells exhibited numerous vacuoles, similar to the cellular morphological changes reported in previous studies (Fig. 1G) [29]. This phenomenon was also similar to the morphological changes observed in HK-2 cells treated with NH_4_Cl (Fig. 1H).

**Fig. 1:**
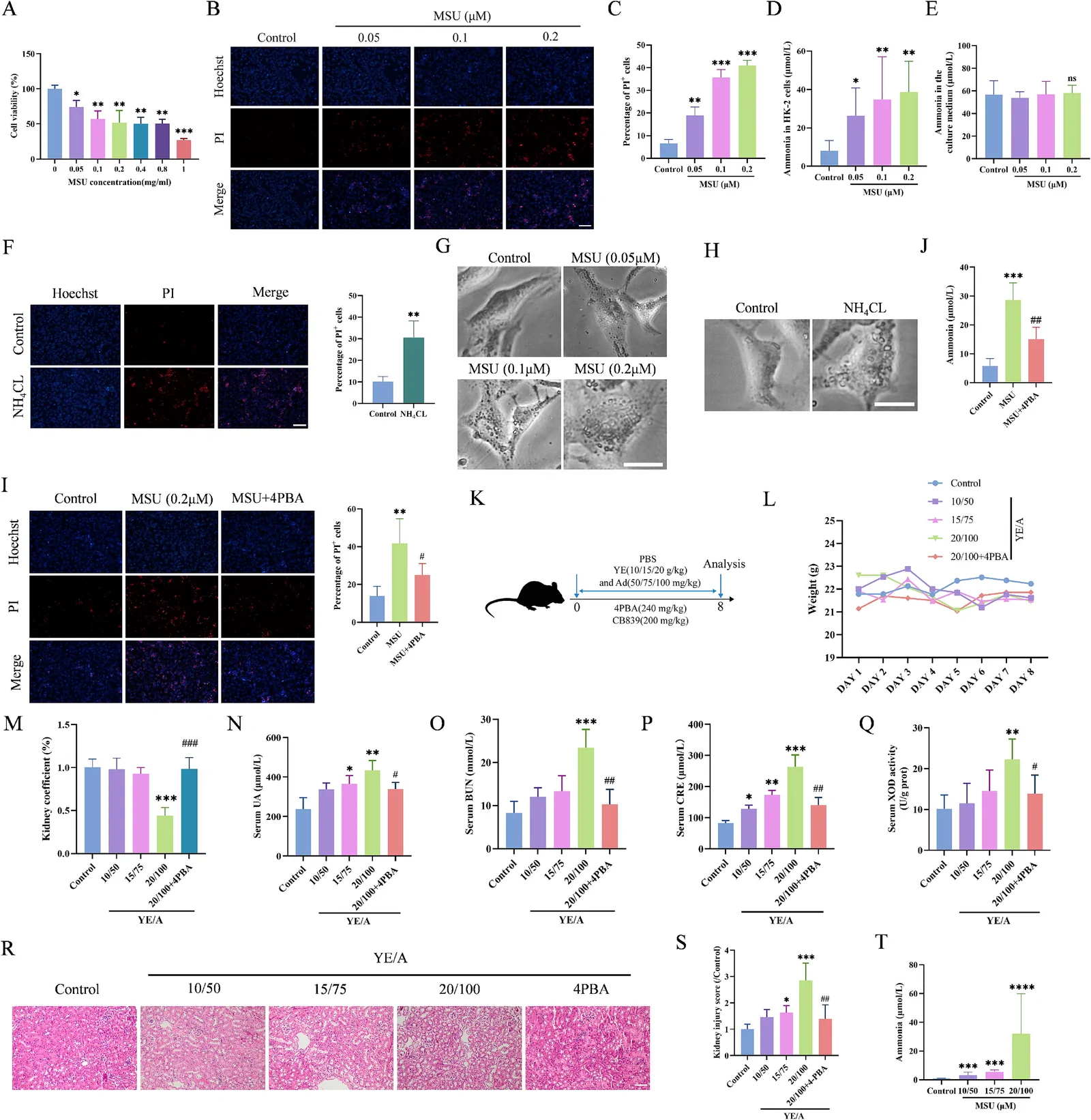
MSU crystals and a high uric acid diet can cause cell vacuolation, ammonia abnormalities, and cell death in vitro and in vivo. (A) Cell viability of HK-2 treated with gradient concentrations of MSU for 24 h by CCK-8 assay. (B-C, F, I) Following staining with PI (2 μg/ml) and Hoechst 33,342 (5 μg/ml) for 10 min, the percentage of PI-positive (red) cells relative to total Hoechst 33342-positive (blue) cells were quantified (Scale bar, 50 μM). (D-E, J) Ammonia levels in renal HK-2 cells and the culture medium. (G-H) Morphological images of HK-2 cells stimulated with MSU and NH_4_Cl for 24 h (Scale bar, 5 μM). (K) In vivo experimental design. Effect of MSU and 4-PBA on body weight (L) and kidney coefficient (M). (N-Q) Serum UA, CRE, BUN, XOD levels in mice kidney. (R) H&E staining from mice (scale bar = 50 μm). (S) Kidney injury scores. (T) Ammonia content in mouse kidney tissue. Data are shown as mean ± SD (n = 3). *P < 0.05, **P < 0.01, ***P < 0.001vs. Control group; ^#^P < 0.05, ^##^P < 0.01, ^###^P < 0.001 vs. MSU group.

To further investigate the role of ammonia, we pre-treated MSU-stimulated HK-2 cells with 4-PBA, which can detoxify ammonia by forming phenylacetylglutamine. This treatment significantly decelerated cell death and ammonia levels (Fig. 1I–J) Specifically, 4-PBA treatment reduced the average cell death rate from 41.71 % to 24.97 % (P < 0.01) and lowered the average ammonia level from 28.67 μmol/L to 15.05 μmol/L (P < 0.05). This confirms that MSU-induced ammonia accumulation is a major cause of cell death in HK-2 cells. These findings suggested that MSU crystals induced ammonia accumulation, leading to vacuolization and cellular demise.

To confirm whether these observations reappear in vivo models, we administered YE and Ad via oral gavage to mice for 8 days to induce a hyperuricemia state (Fig. 1K). While no significant changes in body weight were observed, kidney weight was markedly decreased in the high-dose group, with biochemical markers of hyperuricemia showing significant alterations (Fig. 1L–Q). Kidney tissue exhibited severe damage, including dose-dependent glomerular injury, vacuolar degeneration, and inflammatory cell aggregation (Fig. 1R–S). Notably, ammonia levels in kidney tissues were significantly elevated, particularly in the high-dose group (Fig. 1T). Furthermore, administration of 4-PBA to mice on a high-purine diet dramatically reversed renal damage. This was evidenced by a marked reduction in key pathological and biochemical markers: the kidney injury score decreased by 51.26 % (P < 0.01), alongside significant declines in serum levels of UA (21.99 %, P < 0.05), BUN (55.96 %, P < 0.01), CRE (44.76 %, P < 0.01), and XOD activity (37.87 %, P < 0.05) (Fig. 1L–S). These results suggested that ammonia accumulation played a crucial role in HN, contributing to renal damage and HK-2 cell death. Inhibition of ammonia accumulation effectively mitigated these detrimental effects.

### MSU crystals and a high-purine diet disrupt glutamine metabolism in vivo and in vitro

Ammonia is primarily produced in the kidneys from glutamine via the mitochondrial glutamine synthase 1 enzyme (GLS1). We found that ammonia levels in HK-2 cells increased progressively with different stimulation durations, stabilising at a saturated level after 24 h. Therefore, we selected 24 h as the stimulation duration for MSU in our experiments (Fig. 2A). We also observed that as stimulation time and concentration were gradually increased, GLS1 levels also increased, reaching a stable state at approximately 48 h and 0.1 μM (Fig. 2B–C). Under CB-839 (GLS1 inhibitor) treatment, GLS1 levels were significantly reduced (Fig. 2D). Compared to the MSU group, treatment with CB-839 significantly reduced ammonia levels by 61.45 % (Fig. 2E, p < 0.01) and alleviated HK-2 cell death by 44.49 % (Fig. 2F–G, p < 0.05). To validate the results in vivo, we also measured the same indicators in mice fed a high-purine diet. Similarly, compared to the control group, GLS1 levels were significantly elevated in the modelling group in both WB and IHC analyses, and were markedly alleviated after CB-839 treatment (Fig. 2H–K). Additionally, GLS1 inhibition alleviated renal tissue damage, reflected in a 43.71 % lower kidney injury score (Fig. 2L–M, p < 0.01). Furthermore, GLS1 inhibition alleviated renal uric acid metabolism dysfunction, demonstrated by decreased serum UA (33.16 %, p < 0.01), BUN (48.27 %, p < 0.01), CRE (24.46 %, p < 0.05), and XOD activity (40.27 %, p < 0.01) (Fig. S2B–E). In summary, these results indicated that MSU crystals and a high-purine diet caused disruption of glutamine degradation both in vivo and in vitro, and this disruption was the primary ammonia source inducing renal cell death.

**Fig. 2:**
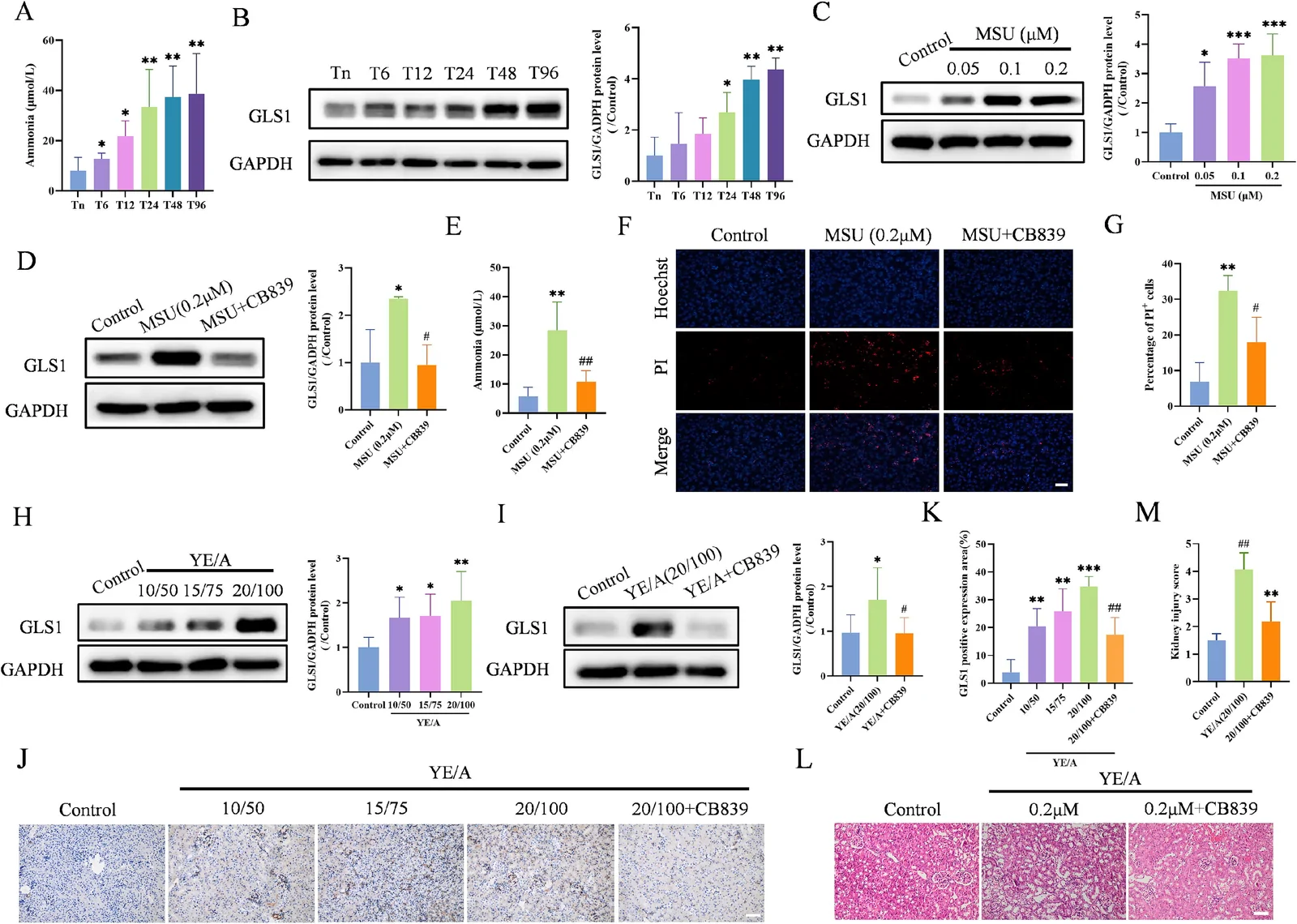
MSU crystals and a high-purine diet led to disturbed intracellular glutamine breakdown in vitro and in vivo. (A, E) Ammonia levels in renal HK-2 cells. (B-D) Western blot of GLS1 in HK-2 cells. (F-G) Cells were stained with PI (2 μg/ml) and Hoechst 33,342 (5 μg/ml) for 10 min (Scale bar, 50 μM). The percentage of PI-positive (red) cells relative to total cells (Hoechst 33342, blue) was analyzed. (H-I) Western blot of GLS1 in mice kidney. (J-K) IHC staining for GLS1 and quantification of the percentage of GLS1-positive area per field of view (scale bar, 50 μM). (L-M) H&E staining and quantification of mouse renal tissue (Scale bar, 50 μM). Data are shown as mean ± SD (n = 3). *P < 0.05, **P < 0.01, ***P < 0.001vs. Control group; ^#^P < 0.05, ^##^P < 0.01 vs. MSU group.

### MSU crystals and a high-purine diet induce excessive ammonia accumulation and lysosomal damage

We next explored the mechanism through which excessive ammonia accumulation, induced by MSU crystals and a high-purine diet, leads to HK-2 cell death. Ammonia, a weak base, exists in both charged (NH_4_^+^) and uncharged (NH_3_) forms. It is capable of crossing cell membranes, including those of lysosomes, and altering the pH within organellar lumens mitochondria utilize potassium channels to expel ammonia, while lysosomes, being acidic organelles, attract alkaline molecules from the cytoplasm. Upon entry into lysosomes, ammonia predominantly binds to protons, a greater proportion than in the cytoplasm, thus reducing the availability of free protons and increasing the lysosomal lumen pH. We observed a gradual increase in ammonium levels in HK-2 lysosomes under high MSU crystal conditions (Fig. 3A). Here, we assume that the accumulation of ammonia in HK-2 lysosomes may also impair lysosomal function. Since lysosomal activity is critically dependent on its internal hydrolytic enzymes, we assessed the activity of Cath-D. Our results revealed that MSU stimulation significantly suppressed Cath-D activity in a dose-dependent manner (Fig. 3B). Furthermore, the expression of V-ATPase B2, a vital component of the proton pump essential for lysosomal acidification, was also substantially downregulated in In vivo and in vitro (Fig. 3C, Fig. S3A–B). In addition, lysosomal acidity is crucial for maintaining normal biological functions and the maturation of autophagic lysosomes [30]. As shown in Fig. 3D–F, lysosomal pH was assessed by Lyso-Tracker Red and Lyso-Sensor Green. In Lyso-Tracker staining, a significant reduction of red dots was observed following MSU crystal stimulation (Fig. 3D, Fig. S3C). Additionally, flow cytometry was used to analyse the average green fluorescence intensity in Lyso-Sensor staining, and the results similarly showed a significant decrease in green fluorescence intensity after MSU crystal stimulation, which indicated that lysosomal pH significantly increased after MSU crystal stimulation (Fig. 3E–F). AO is a lysosomal autofluorescent dye that turns red in lysosomes and green in the cytoplasm, used to detect lysosomal membrane permeability [31]. AO staining results showed that lysosomal membrane permeability significantly increased after MSU treatment (Fig. 3G, Fig. S3D). These results indicated that MSU crystal stimulation caused damage to lysosomal function in HK-2 cells.

**Fig. 3:**
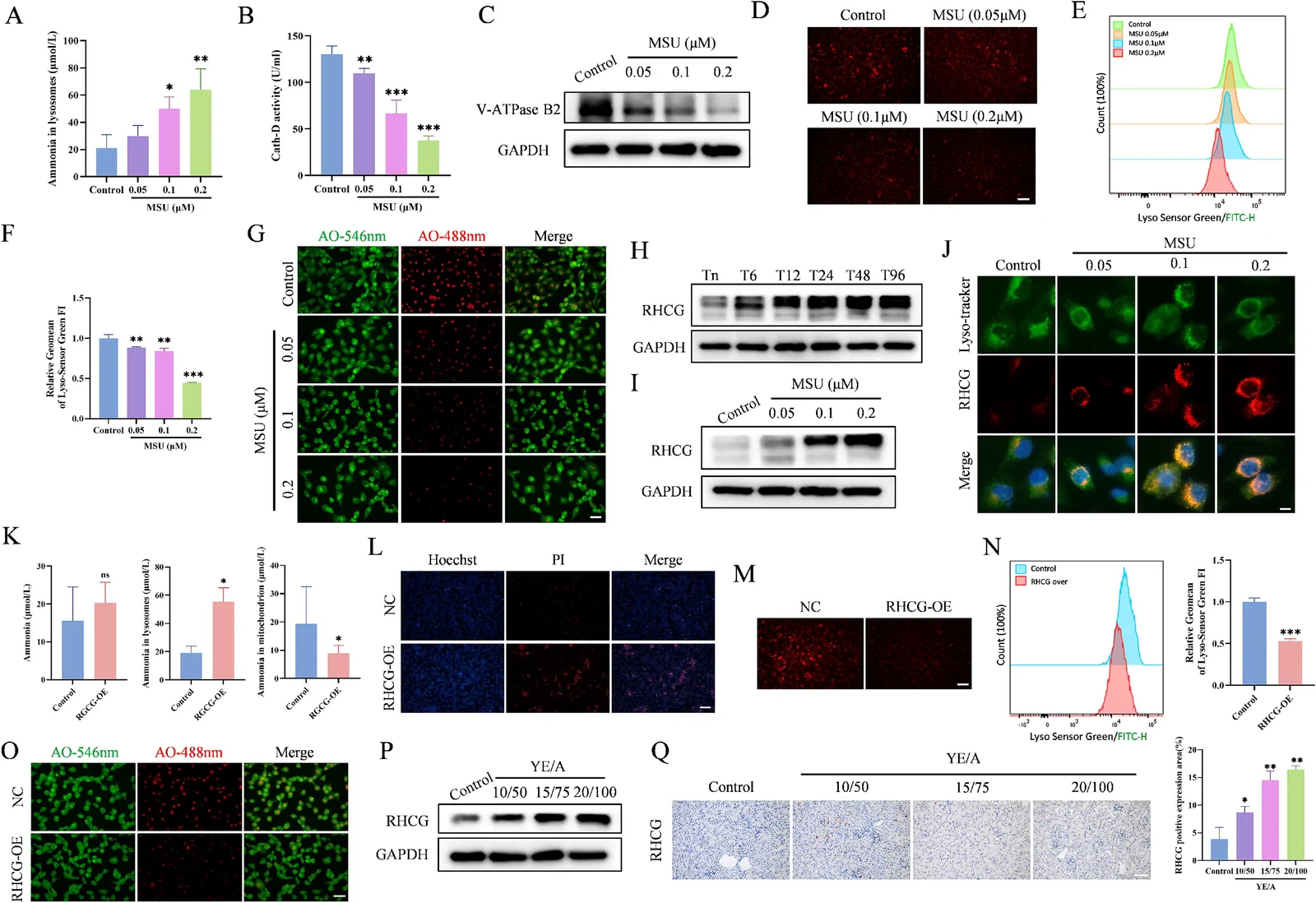
MSU crystals and a high-purine diet cause a large accumulation of ammonia in vitro and in vivo lysosomes to damage lysosomes. HK-2 was transfected with sh-NC (pcDNA3.1) or sh-RHCG (pcDNA3.1(+)-human RHCG) for 48 h (H-L). (A) Ammonia levels in lysosomes of HK-2 cells. (B) The activity of Cath-D in HK-2 cells. (C) Western blot of V-ATPase B2 in HK-2 cells. (D, M) HK-2 stained with LysoTracker RED observed under a microscope (scale bar, 50 μm). (E-F, N) Flow cytometry of LysoSensor GREEN-stained HK-2 treated with MSU to determine the change of lysosomal pH. (G, O) Acetyl orange staining showed that MSU increased the pH of intracellular vesicles and disrupted lysosomes. HK-2 cells were treated with 5 μg/ml acetyl orange at room temperature for 20 min and then washed with PBS. Fluorescence microscopy imaging was performed at a wavelength of 488 nm (scale bar, 50 μm). (H-I) Western blot of RHCG in HK-2 cells. (J) IF staining of RHCG after MSU treatment of HK-2 cells (scale bar, 10 μm). (K) Ammonia levels in HK-2 cells, lysosomes and mitochondrion of HK-2 cells. (L) Cells were stained with PI (2 μg/ ml) and Hoechst 33,342 (5 μg/ml) for 10 min (Scale bar, 50 μM). (P) Western blot of RHCG in mice kidney. (Q) IHC staining for RHCG and quantification of the percentage of RHCG-positive area per field of view (scale bar, 50 μM). Data are shown as mean ± SD (n = 3). *P < 0.05, **P < 0.01, ***P < 0.001vs. Control group.

Current research indicated that RHCG, a key ammonia transporter, facilitates ammonia translocation across organelle membranes, including it could transport ammonia from lysosomes to mitochondria, which is critical for cellular ammonia homeostasis [32]. Through WB experiments, we found that RHCG levels increased in both in vivo and in vitro cells under conditions of MSU crystal formation and a high-purine diet, with increases observed over time and at higher concentrations (Fig. 3H–I, P–Q, Fig. S3E–H). Specifically, RHCG levels in lysosomes were also observed in a concentration-dependent manner (Fig. 3J). Upon RHCG overexpression, the total cellular ammonia levels remained unchanged, whereas lysosomal ammonia levels increased by approximately 191 % and mitochondrial ammonia levels decreased by approximately 53.86 % (Fig. 3K p < 0.05). This result highlighted that the low pH-dependent transport characteristics of RHCG and confirmed that ammonia accumulated extensively in lysosomes via RHCG following MSU crystal stimulation [33]. This RHCG overexpression accelerated HK-2 cell death by 368.89 % (Fig. 3L, Fig. S2J p < 0.001). Consistent with MSU crystal stimulation, HK-2 cells with RHCG overexpression induced lysosomal alkalinization, as demonstrated by a 73.61 % decrease in Lyso-Tracker Red fluorescence (Fig. 3M, Fig. S2K, p < 0.001) and a 46.98 % decrease in Lyso-Sensor Green fluorescence (Fig. 3L, p < 0.001). This alkalinization subsequently led to functional damage, evidenced by a 52.8 % suppression of Cath-D activity (Fig. S3I, p < 0.01) and a 47.29 % increase in lysosomal membrane permeability (Fig. 3O, Fig. S3L). Furthermore, the expression of V-ATPase B2 was substantially downregulated in RHCG OE (Fig. S3M).

By adding 4-PBA to reduce ammonia levels in lysosomes, we found that lysosomal alkalinization, increased membrane permeability and functional damage were alleviated after stimulation. This rescue effect was quantified by a 45.67 % decrease in ammonia levels, alongside the reversal of lysosomal dysfunction: Lyso-Tracker Red fluorescence increased by 41.14 % (p < 0.05), Lyso-Sensor Green fluorescence by 52.05 % (p < 0.01), lysosomal membrane permeability decreased by 56.01 % (p < 0.05), and Cath-D activity was restored by 60.52 % (p < 0.01) (Fig. 4A–E, Fig. S4A). The expression of V-ATPase B2 was also substantially upregulated in 4-PBA-treated HK-2 cells and mice (Fig. S4B–E). Additionally, decrease of ammonia concentrations also lowered RHCG expression in vivo, in vitro, and within lysosomes (Fig. 4F–K). This suggested that inhibiting ammonia in MSU crystal and high-purine diet stimulation alleviated lysosomal damage. To verify whether the significantly elevated ammonia concentration in lysosomes was of mitochondrial origin, we added CB-839 to the experiment. Under GLS1 inhibition, the elevated ammonia levels in lysosomes were significantly reduced by 36.49 % compared to the MSU group (Fig. 5A). Concurrently, lysosomal alkalinization, membrane permeability damage, and functional impairment were alleviated, as demonstrated by increases in Lyso-Tracker Red fluorescence (19.02 %, p < 0.05) and Lyso-Sensor Green fluorescence (51.14 %, p < 0.01), a 30.72 % decrease in membrane permeability (p < 0.05), and a 61.16 % restoration of Cath-D activity (p < 0.01) (Fig. 5B–E, Fig. S4F–J). The expression of V-ATPase B2 was also substantially upregulated in CB-839-treated HK-2 cells and mice (Fig. S4G–J). RHCG expression was reduced in vivo, in vitro, and in lysosomes (Fig. 5F–K). This indicated that ammonia was exported from mitochondria via RHCG and transported to lysosomes. In summary, under stimulation of MSU crystals and a high-purine diet, a large amount of ammonia generated by mitochondrial degradation can be transported and stored in lysosomes via RHCG. Excessive ammonia can lead to alkalisation of lysosomal pH, membrane permeability damage, and functional impairment.

**Fig. 4:**
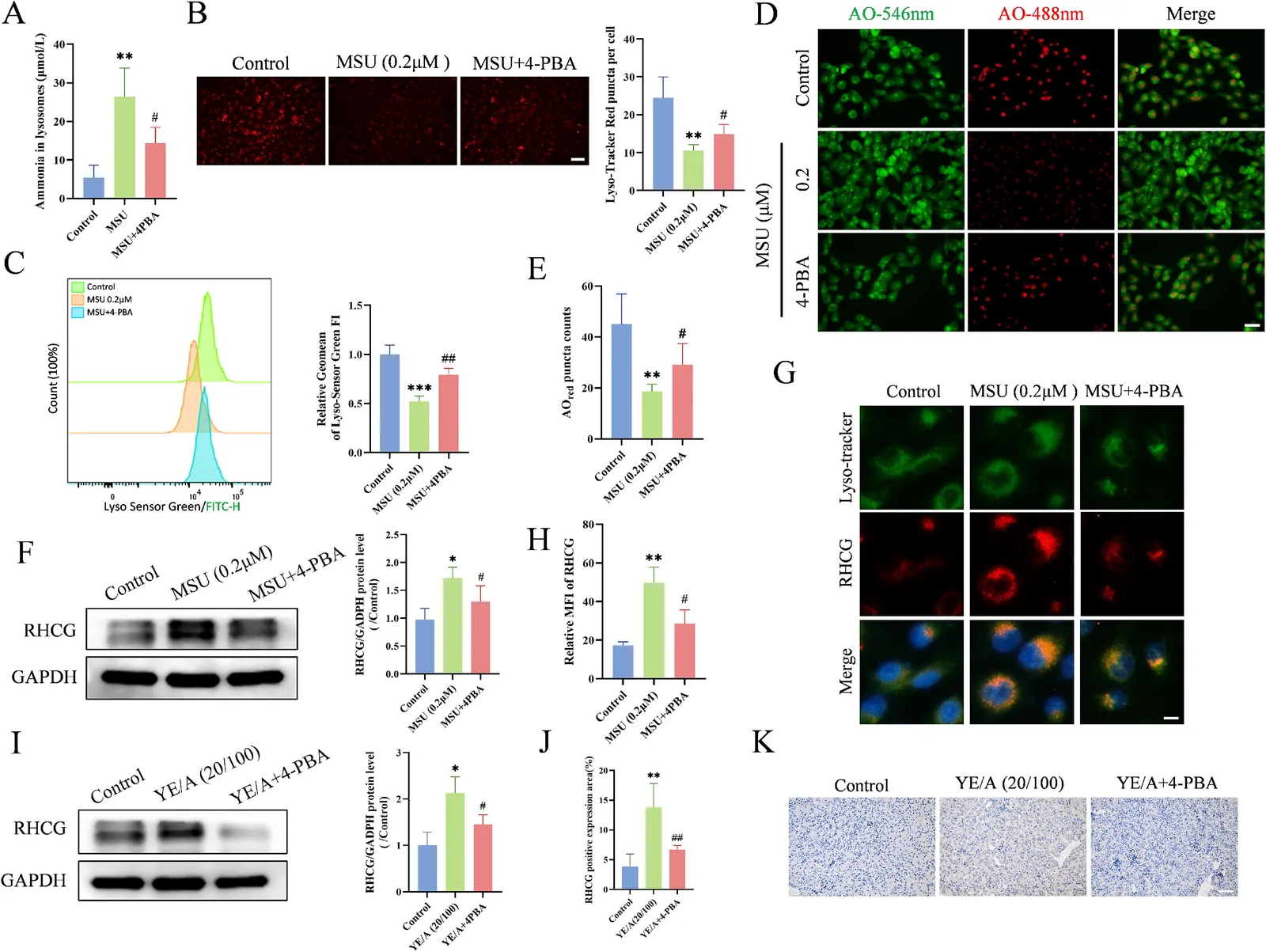
Inhibiting ammonia production alleviates lysosomal damage in HK-2 cells and mice kidneys. (A) Ammonia levels in lysosomes of HK-2 cells. (B) HK-2 stained with LysoTracker RED observed under a microscope (scale bar, 50 μm). (C) Flow cytometry of LysoSensor GREEN-stained HK-2 treated with MSU and 4-PBA to determine the change of lysosomal pH. (D-E) AO staining of HK-2 treated with MSU and 4-PBA (scale bar, 50 μm). (F) Western blot and quantitative analysis of RHCG in HK-2 cells. (G) IF staining of RHCG after MSU and 4-PBA treatment of HK-2 cells (scale bar, 10 μm). (I) Western blot and quantitative analysis of RHCG in mice kidney. (J-K) IHC staining for RHCG and quantification of the percentage of RHCG-positive area per field of view (scale bar, 50 μM). Data are shown as mean ± SD (n = 3). *P < 0.05, **P < 0.01, ***P < 0.001vs. Control group; ^#^P < 0.05, ^##^P < 0.01, ^###^P < 0.001 vs. MSU group.

**Fig. 5:**
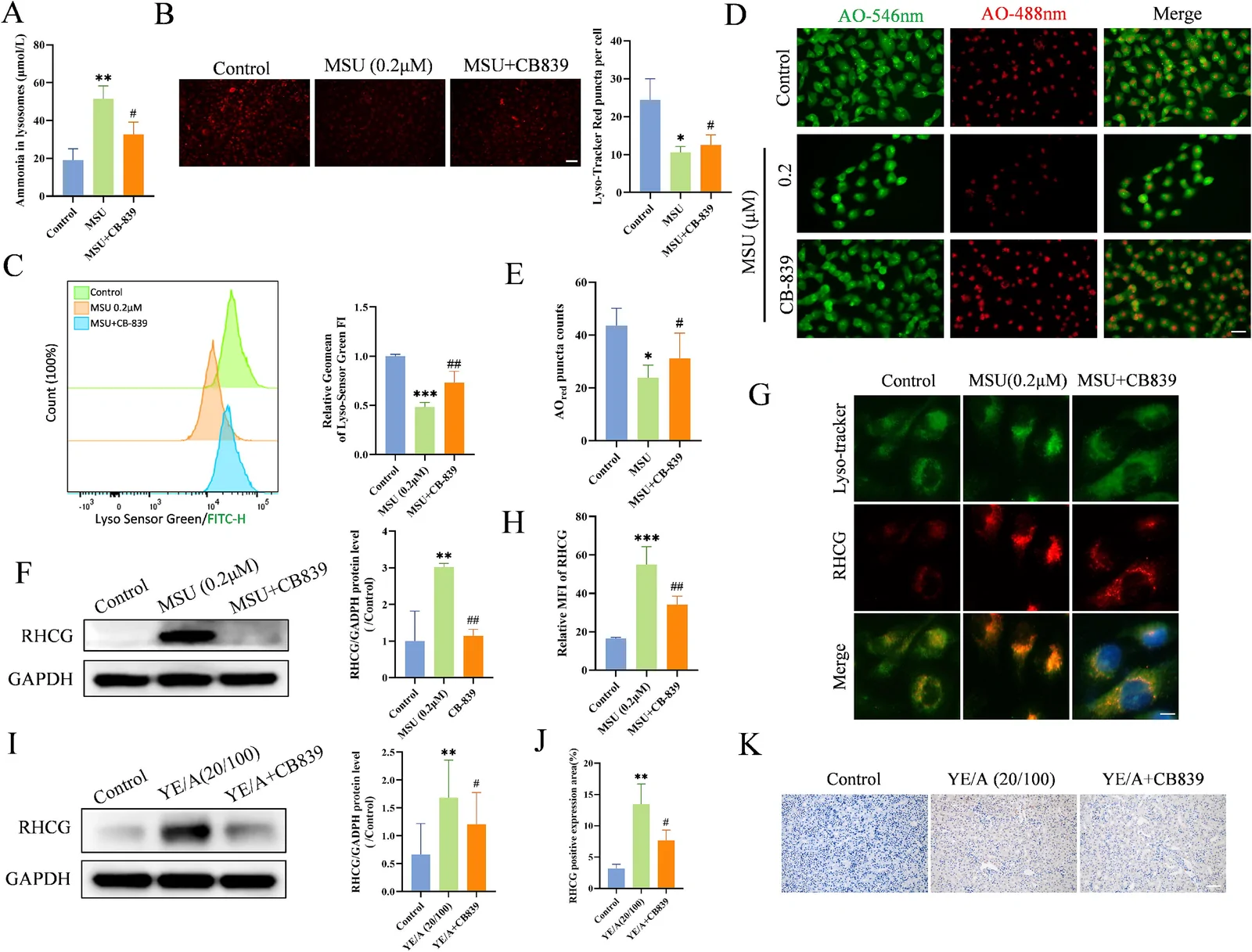
MSU crystals and a high-purine diet stimulate the accumulation of ammonia in lysosomes, which is derived from mitochondria. (A) Ammonia levels in lysosomes of HK-2 cells. (B) HK-2 stained with LysoTracker RED observed under a microscope (scale bar, 50 μm). (C) Flow cytometry of LysoSensor GREEN-stained HK-2 treated with MSU and CB839 to determine the change of lysosomal pH. (D-E) AO staining of HK-2 treated with MSU and CB839 (scale bar, 50 μm). (F) Western blot and quantitative analysis of RHCG in HK-2 cells. (G) IF staining of RHCG after MSU and CB839 treatment of HK-2 cells (scale bar, 10 μm). (I) Western blot and quantitative analysis of RHCG in mice kidney. (J-K) IHC staining for RHCG and quantification of the percentage of RHCG-positive area per field of view (scale bar, 50 μM). Data are shown as mean ± SD (n = 3). *P < 0.05, **P < 0.01, ***P < 0.001vs. Control group; ^#^P < 0.05, ^##^P < 0.01, ^###^P < 0.001 vs. MSU group.

### Ammonia retention induces mitochondrial damage in the kidneys

The results presented above suggested that ammonia was transferred from the mitochondria to the cytoplasm, where it subsequently accumulated in lysosomes at high concentrations, leading to cellular damage. Upon lysosomal damage, the increase in pH prevented further ammonia entry into the lysosomes, resulting in its retention within the mitochondria. Consistent with this, elevated mitochondrial ammonia levels were observed in HK-2 cells following stimulation with MSU crystals (Fig. 6A). This sustained increase in ammonia concentration within mitochondria may consume protons to produce ammonium, disrupting the proton balance and redox homeostasis within mitochondria, thereby causing mitochondrial damage. Experiments confirmed that the MMP in HK-2 cells did indeed decrease after stimulation (Fig. 6B–C); and ROS, a marker of oxidative stress, also significantly increased (Fig. 6D). More notably, mitochondrial matrix swelling, including membrane and cristae structure damage and reduced cristae number, was observed in both in vitro and in vivo (Fig. 6E and K).

**Fig. 6:**
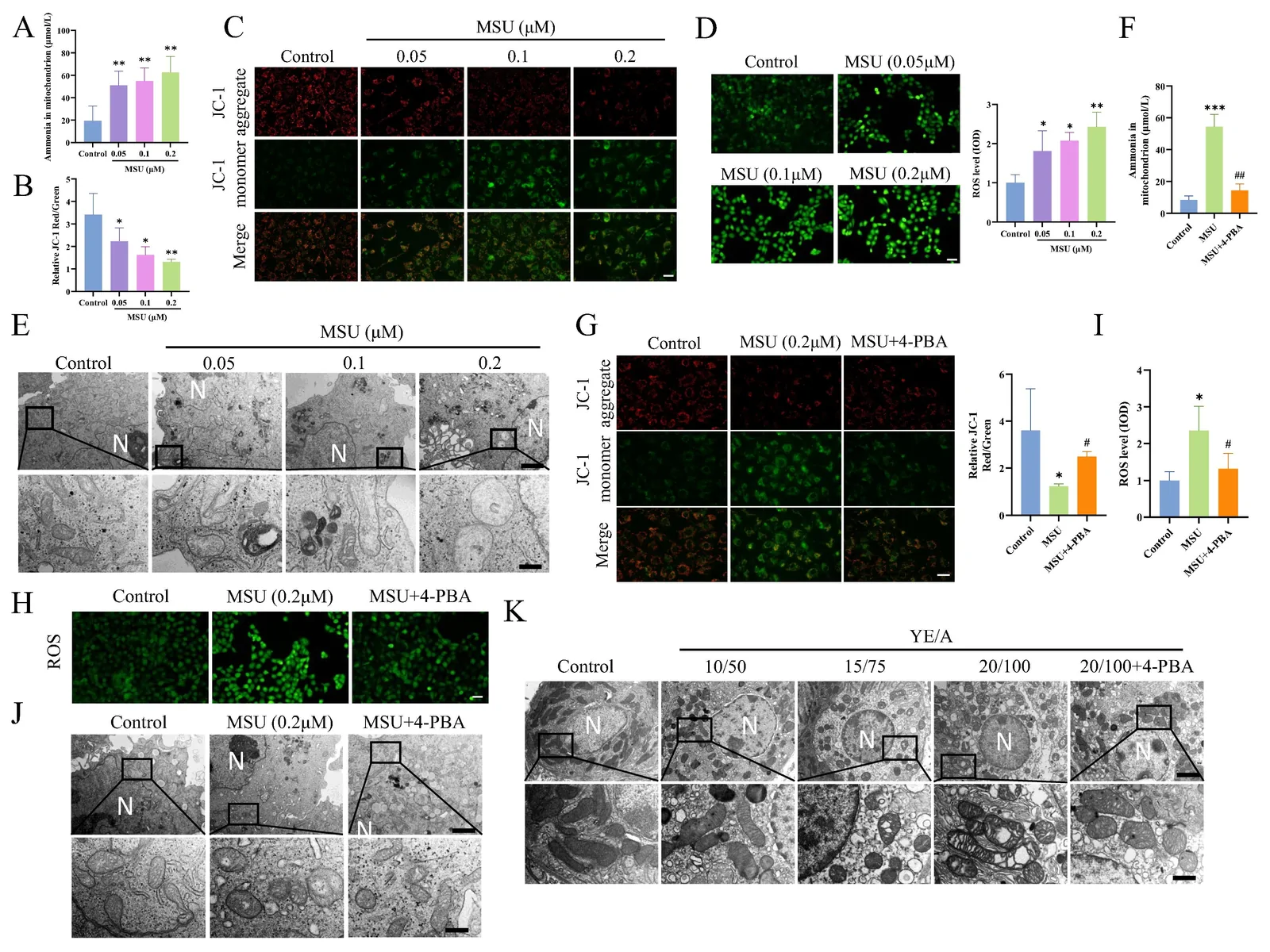
Ammonia retention causes mitochondrial damage. (A, F) Ammonia levels in mitochondria of HK-2 cells. (B-C, G) The HK-2 cell were stained with JC-1 to measure MMP, and the MMP were calculated by the ratio of red/green (scale bar, 50 μm). (D, H-I) ROS production was measured by DCFH-DA staining in HK-2 cells (scale bar, 50 μm), statisitics of the relativity of ROS fluorescence. (E, J-K) Representative images of mitochondria cristae by transmission electron microscopy in HK-2 cells and renal tissues (scale bar, 2 μM and 500 nM). Data are shown as mean ± SD (n = 3). *P < 0.05, **P < 0.01, ***P < 0.001vs. Control group; ^#^P < 0.05, ^##^P < 0.01 vs. MSU group.

To confirm that the observed mitochondrial damage was caused by ammonia retention, we administered 4-PBA to reduce ammonia levels in vivo. Under these conditions, the damaged mitochondria were rescued, marked by a 73.64 % drop in mitochondrial ammonia (Fig. 3Fp < 0.01), a recovery of membrane potential (100.33 % decrease in relative JC-1 red/green, p < 0.05), and a 44.18 % decrease in ROS levels (p < 0.05) (Fig. 6G–I), while matrix swelling and cristae structural damage were alleviated (Fig. 6J).

### MSU crystals and a high-purine diet induce mitochondrial autophagy flux blockage in vivo and in vitro

Mitochondrial autophagy typically facilitates the removal of damaged mitochondria. However, our results demonstrated a significant accumulation of damaged mitochondria in cells following stimulation with MSU crystals and a high-purine diet, suggesting a defect in mitochondrial clearance. To investigate the impact on autophagy, we assessed the expression of autophagy markers, including LC3 and the selective autophagy substrate SQSTM1/p62, using WB analysis (Fig. 7A–D and H). We observed that autophagy appeared to be impaired in stimulated cells, despite the formation of autophagosomes, as indicated by the upregulation of p62 and LC3-II. However, the autophagy flux seemed to be disrupted, as evidenced by the sustained elevation of p62 and LC3-II, suggesting that MSU crystal stimulation and a high-purine diet inhibit autophagy flux and the degradation of autophagic vesicles. This elimination of autophagy flux is primarily caused by lysosomal alkalinization, which impairs hydrolase activity, thereby blocking autophagolysosome degradation-a late-stage critical step in autophagy-resulting in undigested autophagosome accumulation [34]. In the experiment, we found that LC3-II and p62 co-localisation with mitochondria after in vivo and in vitro stimulation can serve as markers of mitochondrial phagocytosis [35]. Consistent with previous results, we observed a significant increase in LC3 fluorescence spots, co-localisation with mitochondria, and extensive expression in tissues (Fig. 7E and J). To visualise changes in intracellular autophagy flux, we observed the subcellular structures of HK-2 and renal tissue cells treated with in vivo and in vitro modelling using transmission electron microscopy (Fig. 7F, G and I). Cytoplasmic autophagic lysosomes, which result from the fusion of autophagosomes and lysosomes, showed a significant increase in number following stimulation. The marked accumulation of sealed double-membrane autophagosomes and autophagic lysosomes indicates failed autophagic degradation, thereby confirming that MSU crystal stimulation and a high-purine diet block autophagic flux.

**Fig. 7:**
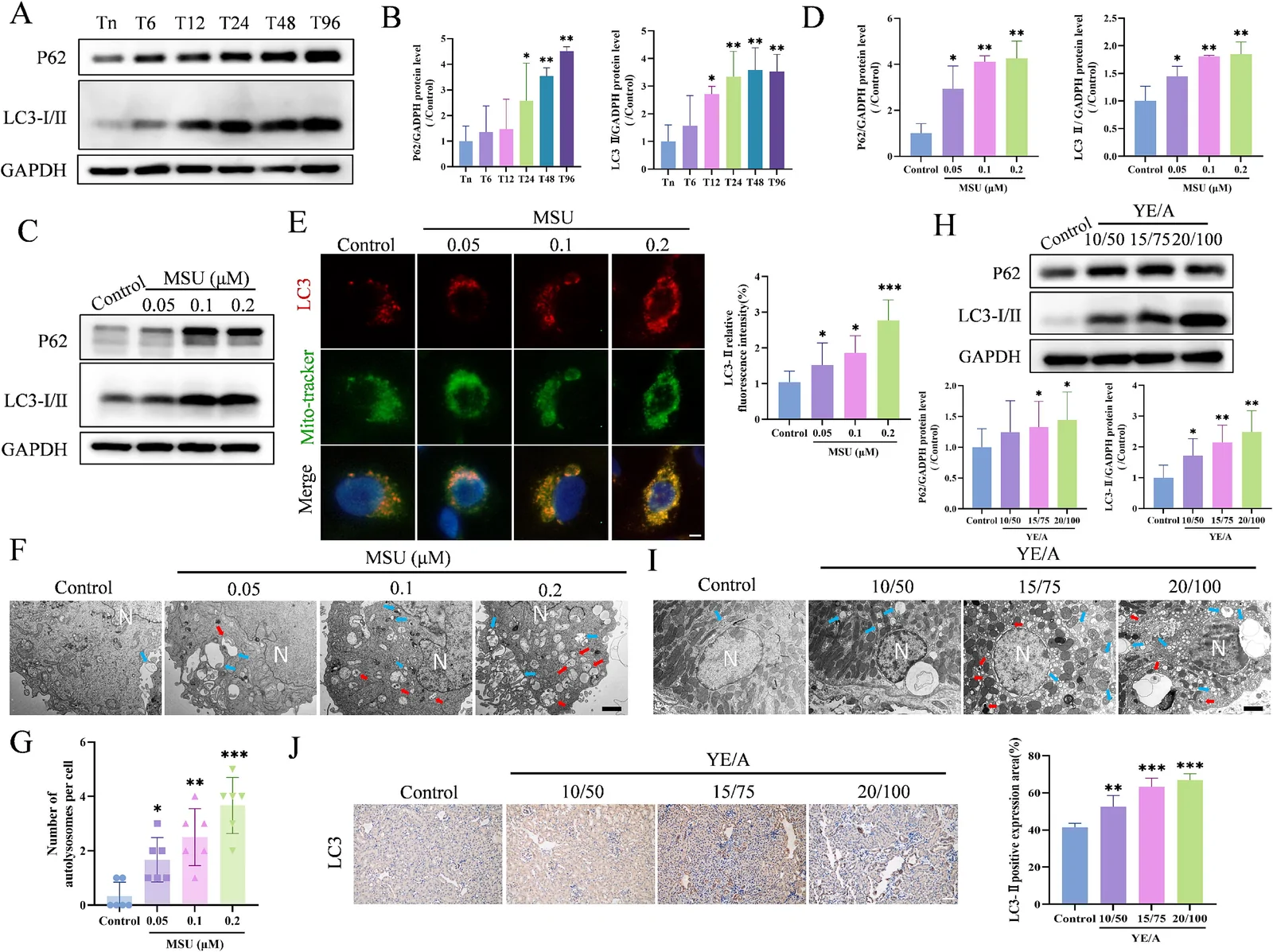
MSU crystals and a high-purine diet induced mitochondrial autophagy flux blockage. (A-B, C-D) Western blot and quantitative analysis of P62 and LC3-Ⅰ/Ⅱ in HK-2 cells. (E) IF staining of LC3 after MSU treatment of HK-2 cells (scale bar, 10 μm). (F) TEM images and (G) the quantification of autophagy-related structures in HK-2 treated with MSU (Scale bar, 2 μm. N: nucleus; Blue arrow: autophagosome; Red arrow: autolysosomes). (H) Western blot and quantitative analysis of P62 and LC3-Ⅰ/Ⅱ in mice kidney. (I) TEM images of autophagy-related structures in renal tissues (Scale bar, 2 μm. N: nucleus; Blue arrow: autophagosome; Red arrow: autolysosomes). (J) IHC staining for LC3 and quantification of the percentage of LC3-positive area per field of view (scale bar, 50 μM). Data are shown as mean ± SD (n = 3). *P < 0.05, **P < 0.01, ***P < 0.001vs. Control group.

The above results indicated that the blockage of autophagy flux induced by MSU crystals and a high-purine diet occurred after the formation of LC3-containing autophagosomes and before autophagolysosomal degradation. To further validate this, we used early and late autophagy inhibitors 3-MA and BafA1, which inhibit PI3K-initiated autophagosome formation and lysosomal membrane H-ATPase respectively to pre-treat the cells [36,37]. As shown by WB (Fig. 8A and I), IF analysis (Fig. 8B and F; Fig. S5A), and IHC (Fig. 8M, Fig. S5C), 3-MA pretreatment increased MSU crystal- and high-purine diet-induced LC3-II expression, with a corresponding reduction in LC3 immunofluorescence intensity (by 45.36 %, p < 0.05) and IHC-positive area intensity (by 26.17 %, p < 0.01). In contrast, Baf A1 pretreatment had no effect on induced LC3 expression (Fig. 8E, F, J and M; Fig. S5B and C). Electron microscopy revealed that 3-MA reduced the number of single-membrane autophagic lysosomes and autophagosomes induced by stimulation, with an average reduction of approximately 2.83 autolysosomes per cell (p < 0.01), while Baf A1 had minimal effect (Fig. 8C–D, G–H and L). Similar autophagic flux blockage was observed in NH4Cl-treated HK-2 cells (Fig. S5D). These data confirm that MSU crystals and a high-purine diet induce late-stage blockage of the autophagic flux (Fig. 8K). In summary, MSU crystals and a high-purine diet block late-stage autophagic lysosomal degradation, preventing the accumulation of ammonia in damaged mitochondria from being cleared via autophagy, thereby inducing cell death.

**Fig. 8:**
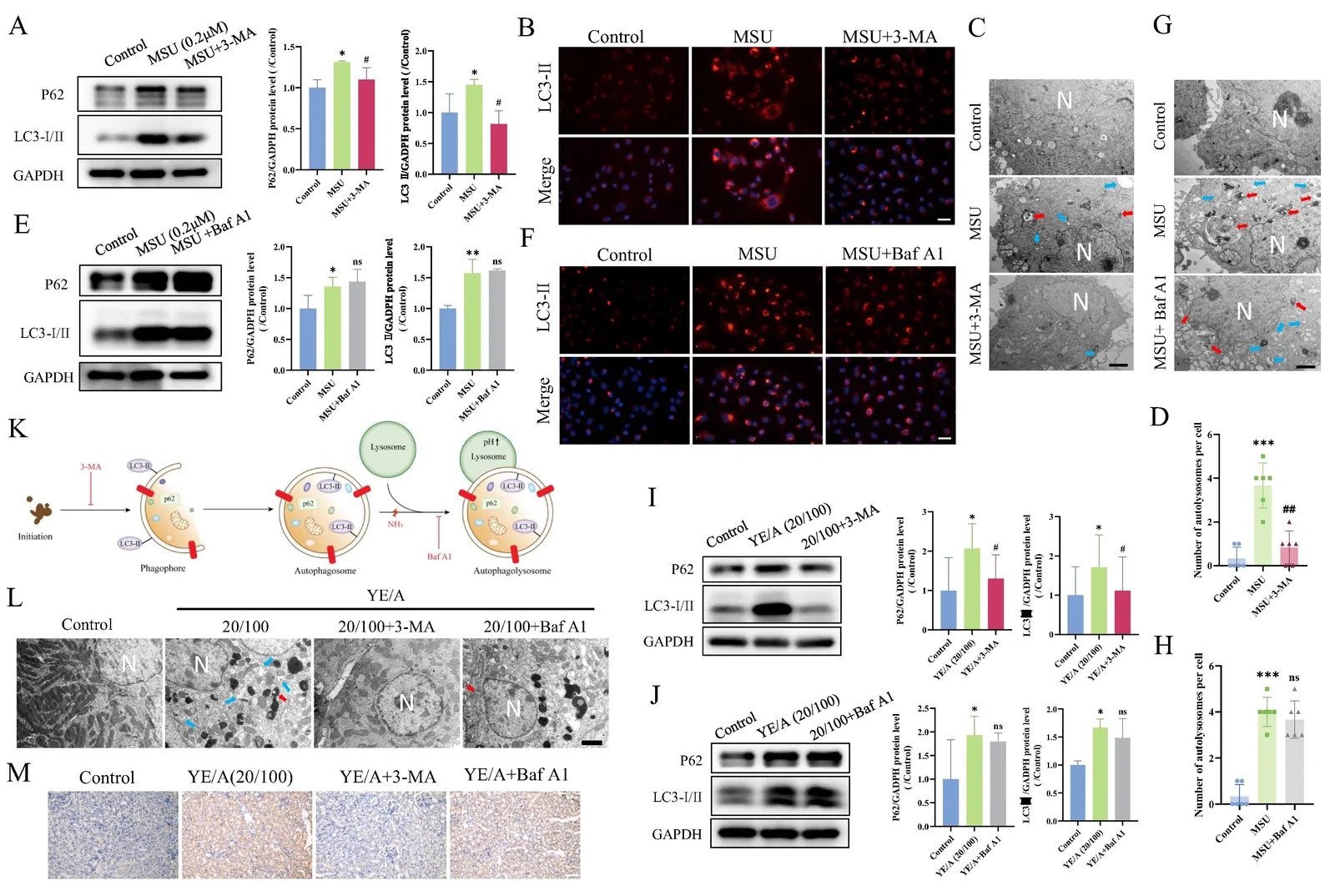
MSU crystals and a high-purine diet can inhibit late-stage degradation of the autophagy flux. (A, E) Western blot and quantitative analysis of P62 and LC3-Ⅰ/Ⅱ in HK-2 cells. (B, F) IF staining of LC3-II and quantification of its average fluorescence intensity per cell after treatment of HK-2 cells with MSU and 3-MA or Baf A1 (scale bar, 50 μm). (C, G) TEM images and (D, H) the quantification of autophagy-related structures in HK-2 treated with MSU and 3-MA or Baf A1 (Scale bar, 2 μm. N: nucleus; Blue arrow: autophagosome; Red arrow: autolysosomes). (I-J) Western blot and quantitative analysis of P62 and LC3-Ⅰ/Ⅱ in mice kidney. (K) Schematic diagram showing major findings in this figure. (L) TEM images of autophagy-related structures in renal tissues (Scale bar, 2 μm. N: nucleus; Blue arrow: autophagosome; Red arrow: autolysosomes). (M) IHC staining for LC3 and quantification of the percentage of LC3-positive area per field of view (scale bar, 50 μM). Data are shown as mean ± SD (n = 3). *P < 0.05, **P < 0.01, ***P < 0.001vs. Control group; ^#^P < 0.05, ^##^P < 0.01, ^###^P < 0.001 vs. MSU group.

A comprehensive summary of these key experimental findings is provided in Supplementary Table S1.

## Discussion

During evolution, cells metabolise carbohydrates, lipids, and amino acids to produce ATP for energy use. In these processes, ROS produced by the oxidation of carbohydrates and lipids and ammonia produced by the deamination of amino acids are inevitably generated. These two toxic by-products may lead to cell death [38]. There is extensive literature documenting ROS-related renal cell death (including apoptosis, pyroptosis, and ferroptosis) in HN [39]. However, ammonia-related cell death has not yet been assessed. Therefore, this study aimed to explore whether the disorder of ammonia metabolism is an independent and key driver of cell death in HN and its underlying mechanism. In this study, we explored whether ammonia-induced cell death occurs in damaged renal epithelial cells under hyperuricemic conditions. Previous studies have shown that ammonia is produced in mitochondria and transported from mitochondria to the cytoplasm, then further into lysosomes [29]. Our study indicated that under prolonged hyperuricemic conditions, the degradation of glutamine was accelerated to alleviate damage and produce sufficient ATP. The excessive production and transport of ammonia lead to lysosomal alkalisation and subsequent mitochondrial ammonia retention, as well as defects in the clearance of damaged mitochondria, ultimately resulting in cell death.

HN refers to the phenomenon of renal functional damage occurring in a high UA environment. During HN, numerous cellular and molecular events occur, including apoptosis, inflammatory infiltration, necrosis, and epithelial-mesenchymal transition (EMT) [6,40,41]. Some studies suggest that ammonia can damage developing T-eff cells through a novel mechanism. Specifically, excessive ammonia secreted by mitochondria leads to alkalisation of lysosomes, thereby disrupting their acidic environment. This alkalising effect inhibits the normal function of lysosomes, preventing them from effectively degrading metabolic waste within cells, leading to the accumulation of intracellular waste and ultimately causing cell death [29]. These studies have primarily focused on the liver and immune cells, with no similar investigations conducted in the kidneys.

Our study initially confirmed that ammonia levels were significantly and persistently elevated in renal cells within the MSU stimulation and high-purine diet-induced HN models (Fig. 1), providing a premise for investigating ammonia toxicity. To explore the source of ammonia, we found that the expression of GLS1, a key enzyme in mitochondrial glutamine metabolism, was significantly upregulated (Fig. 2), suggesting that metabolic reprogramming is the root cause of excessive ammonia production. Subsequently, we focused on the toxic targets of ammonia. We observed significant alkalinization and functional impairment of lysosomes (Fig. 3), alongside a marked decrease in mitochondrial membrane potential accompanied by ROS accumulation (Fig. 6). A key finding was that overexpression of the ammonia transporter RHCG markedly exacerbated these injuries and cell death (Fig. 3), whereas inhibition of GLS1 significantly alleviated the damage (Fig. 5). Our study confirmed that RHCG, as a key ammonia transporter, mediates ammonia translocation into lysosomes, further exacerbating cellular damage under MSU and high-purine diet conditions. Based on this series of dose-dependent and intervention experiments, we can infer that GLS1 and RHCG are not merely “involved”, but rather constitute core rate-limiting components in the “ammonia production-ammonia transport-organelle damage” pathway. Further analysis revealed that mitochondrial damage involved the destruction of MMP and the accumulation of ROS. It has been reported that ROS triggers autophagy to clear damaged mitochondria from the cell to maintain cell survival [42,43]. In autophagy-deficient cells, the accumulation of ROS and damaged mitochondria ultimately leads to cell death [44].

Therefore, we aimed to further investigate the changes in the signalling pathways of cellular autophagy both in vitro and in vivo. The role of autophagy in this process is multifaceted and dichotomous. In the early stages of cellular damage, autophagy can degrade damaged mitochondria, regulate mitochondrial numbers, and maintain the stability of cellular energy metabolism. However, in the later stages of cellular damage, excessive autophagy can lead to cell death [45]. Unlike simple autophagy activation, our data demonstrate that MSU crystals stimulation and a high-purine diet led to concurrent substantial accumulation of both autophagosomes and impaired autolysosome formation (Fig. 7). This distinctive pattern strongly suggests a blockade of autophagic flux at the lysosomal degradation stage, rather than an activation of autophagosome formation. The failure to promptly clear damaged mitochondria further exacerbate oxidative stress, ultimately leading to cell death. Consequently, these damaged renal epithelial cells underwent cell death, consistent with previous studies [46,47].

To precisely pinpoint the stage at which autophagic flux is blocked, we employed autophagy inhibitors. Notably, the early-stage inhibitor 3-MA partially alleviated the accumulation of autophagosomes and cell death, whereas the late-stage inhibitor Baf A1 had no such effect (Fig. 8). This indicates that the blockade in the autophagy pathway primarily occurs after autophagosome formation but before autolysosomal degradation. Combined with our previous findings of lysosomal alkalinization and functional impairment (Fig. 3), these data collectively confirm that lysosomal dysfunction is the direct cause of failed autolysosomal degradation. Beyond these observations, our discovery of autophagic flux blockade prompts us to consider the upstream signaling pathways that may coordinate this dysfunction. Existing literature indicates that inhibition of mTORC1 is a classical signal for autophagy activation, and MSU crystals have been shown to inhibit mTORC1 activity [48]. This is highly relevant to our model, as mTORC1 inhibition is a well-established trigger for autophagosome initiation, which could explain the observed increase in autophagosomes following MSU crystals stimulation (Fig. 7). This provides a plausible upstream signaling explanation for our core findings. Future studies are needed to directly measure mTORC1 activity to validate this hypothesis, but this speculation offers a exploratory direction for understanding how chronic metabolic injury in HN disrupts cellular autophagy.

Autophagy is typically divided into an early stage of autophagosome formation and a late stage of degradation mediated by lysosomal fusion [49]. We found that the early autophagy inhibitor 3-MA partially alleviated autophagy flux inhibition in the environment of HN, but the late autophagy inhibitor Baf A1 had no effect (Fig. 8). This suggested that the blockade of this autophagy pathway primarily occurred after autophagosome formation and before autophagolysosomal degradation, indicating that it inhibited autophagolysosomal degradation. Combining previous studies on lysosomal alkalisation and functional damage, we speculated that lysosomal damage led to insufficient acidity in lysosomes to degrade autophagic substrates within autophagolysosomes, thereby obstructing the late stages of the autophagy pathway.

In summary, our study demonstrated that ammonia-induced cell death occured in the context of chronic HN. Mitochondrial glutamine metabolism was disrupted, accompanied by increased ammonia secretion. Large amounts of ammonia were transported to lysosomes via RHCG, leading to alkalisation of the lysosomal environment. Critically, this lysosomal alkalinization impairs hydrolase activity, thereby blocking autophagolysosome degradation-a late-stage critical step in autophagy-resulting in undigested autophagosome accumulation. This in turn causes ammonia accumulation and damage within mitochondria. Ultimately, this disruption resulted in late-stage blockage of the autophagy pathway, leading to cell death. The long-term accumulation of ammonia leads to cellular dysfunction, resulting in cellular damage and ultimately cell death. Therefore, inhibiting ammonia production may serve as an effective strategy to alleviate HN. Additionally, suppressing the upregulation of GLS1 or promoting lysosomal acidification can also mitigate ammonia-induced cell death, both of which contribute to slowing the progression of HN.

Our study proposes a shift in understanding HN pathogenesis, moving beyond the established paradigms of direct urate crystal toxicity and consequent inflammatory responses. While previous research has rightly focused on crystal-induced physical damage and inflammation [20], our findings reveal a parallel, metabolism-centric pathway that operates even in the absence of overt crystal deposition. The core of this innovation lies in identifying ammonia as a novel, soluble metabolic mediator of injury, distinct from the solid-phase urate crystals. This ammonia-induced cell death pathway is metabolically initiated by glutamine decomposition. It then culminates in lysosomal dysfunction and autophagic flux blockade, thereby explaining a form of cellular stress that is fundamentally metabolic in nature. Importantly, this mechanism can elucidate pathological phenomena not fully accounted for by existing theories. For instance, the preferential injury to renal tubular cells, particularly in the S3 segment of the proximal tubule which is rich in mitochondria, may be attributed to their high metabolic activity and consequent susceptibility to ammonia-induced metabolic disruption and mitochondrial damage [50]. Thus, our model does not negate previous theories but rather complements them by introducing a crucial metabolic dimension, offering a more integrated explanation for the complex pathology of HN.

Our study also has several limitations. Firstly, the animal models used represent a relatively subacute injury, which may not fully recapitulate the chronic nature of human HN. Future studies employing more appropriate chronic disease models or examining human kidney tissues are warranted. Secondly, although we speculate on the involvement of upstream signaling pathways such as mTORC1, direct evidence is lacking, which presents an important direction for subsequent investigation. Finally, our research primarily focused on HK-2 cells, whether similar mechanisms operate in other renal cell types remains to be elucidated.

## Conclusion

In summary, our study demonstrates that chronic hyperuricemia triggers a novel ammonia mediated cell death pathway in renal cells. This pathway is initiated by GLS1-driven ammoniagenesis, followed by RHCG-mediated ammonia transport into lysosomes, resulting in alkalinization and functional impairment. Crucially, we have clarified that lysosomal alkalinization impairs hydrolase activity, thereby blocking autophagolysosome degradation and leading to the accumulation of undigested autophagosomes and damaged mitochondria. This ammonia-induced autophagic flux blockade represents the fundamental mechanism driving cell death. Consequently, our findings identify the targeting of ammonia production (via GLS1 inhibition) or the restoration of lysosomal function as promising therapeutic strategies for mitigating HN. By elucidating key steps in the cascade from metabolic disruption to lysosomal dysfunction and failed quality control, our work contributes to a more refined pathophysiological framework for understanding HN and pinpoints specific molecular targets for future investigation.

## Compliance with ethics requirements

The animal study protocol was approved by the Animal Care and Use Committee of the First Hospital of Jilin University (Approval No. 11569). The study adhered to the guidelines set by the committee.

## Declaration of competing interest

The authors declare that they have no known competing financial interests or personal relationships that could have appeared to influence the work reported in this paper.
