# Supplementary material for: Hyperuricemia increases susceptibility to chronic kidney injury exacerbation via autophagic flux blockade-mediated ammonia death pathway
Source: J Adv Res. 2025 Nov 7;88:743–57. doi: 10.1016/j.jare.2025.11.003 (PMC13669550; doi:10.1016/j.jare.2025.11.003)
Supplement: Supplementary file 1 [file mmc1.docx]

**Hyperuricemia increases susceptibility to chronic kidney injury exacerbation via autophagic flux blockade-mediated ammonia death pathway**

**Supplementary file**

**Fig. S1 Schematic Illustration of the Experimental Design.** Schematic overview of the experimental design and procedures employed in this study. A comprehensive description of each procedure is provided in the corresponding Methods subsections.

**Fig. S2 Supplementary data related to Figure 2.** (A)Molecular structure of MSU. (B-E) Serum UA, CRE, BUN, XOD levels in mice kidney. Data are shown as mean ± SD (n = 3). *P < 0.05, **P < 0.01, ***P < 0.001vs. Control group; #P < 0.05, ##P < 0.01 vs. MSU group.

**Fig. S3 Supplementary data related to Figure 3.** (A) The protein expression level of V-ATPase B2 in HK-2 cells. (B, M) The protein expression level and quantitative analysis V-ATPase B2 in cells and mice. (C, K) Quantitative analysis LysoTracker RED staining. (D, L) Quantitative analysis of AO staining of HK-2. (E-G) Quantitative analysis of WB. (H) IF quantitative analysis. (I) The activity of Cath-D in HK-2 cells. (J) The percentage of PI-positive (red) cells relative to total cells (Hoechst 33342, blue) was analyzed. Data are shown as mean ± SD (n = 3). *P < 0.05, **P < 0.01, ***P < 0.001vs. Control group.

**Fig. S4 Supplementary data related to Figure 4 and Figure 5.** (A, F) The activity of Cath-D in HK-2 cells. (B-E, G-H) The protein expression level and quantitative analysis V-ATPase B2 in cells and mice. Data are shown as mean ± SD (n = 3). *P < 0.05, **P < 0.01, ***P < 0.001vs. Control group; #P < 0.05, ##P < 0.01 vs. MSU group.

**Fig. S5 Supplementary data related to Figure 8 and the effect of NH_4_CL on HK-2 autophagy.** (A-B) IF quantitative analysis. (C) IHC quantitative analysis. (D) TEM images of autophagy-related structures in renal tissues (Scale bar, 2 μm. N: nucleus; Blue arrow: autophagosome; red arrow: autolysosomes). Data are shown as mean ± SD (n = 3). **P < 0.01, ***P < 0.001vs. Control group; #P < 0.05, ##P < 0.01 vs. MSU group.
