# Supplementary material for: Hyperuricemia increases susceptibility to chronic kidney injury exacerbation via autophagic flux blockade-mediated ammonia death pathway
Source: J Adv Res. 2025 Nov 7;88:743–57. doi: 10.1016/j.jare.2025.11.003 (PMC13669550; doi:10.1016/j.jare.2025.11.003)
Supplement: Supplementary file 2 [file mmc2.docx]

Table 1 Summary of Key Experimental Findings

| Experimental Group | Key Metrics | Change vs. Control | Change vs. MSU/ High purine diet Model | Figure Reference |
| --- | --- | --- | --- | --- |
| MSU/ High purine diet Model (High dose) | Ammonia levels | ↑↑ | - | Fig. 1D |
|  | Lysosomal/ Mitochondrial ammonia | ↑↑/↑↑ | - | Fig. 3A, Fig. 6A |
|  | Cell death/Renal injury | ↑↑↑/↑↑↑ | - | Fig. 1B-C/R-S |
|  | Serum UA/BUN/CRE/XOD | ↑↑/↑↑↑/↑↑↑/↑↑ | - | Fig. 1N-O |
|  | GLS1 expression (cells/tissues/IHC) | ↑↑↑/↑↑/↑↑↑ | - | Fig. 2C/H/J-K |
|  | Cath-D activity | ↓↓↓ | - | Fig. 3B |
|  | V-ATPase B2 expression (cells/tissues) | ↓↓/↓↓↓ | - | Fig. 3C, Fig. S3A-B |
|  | Lyso-Tracker Red | ↓↓↓ | - | Fig. 3D, Fig. S3C |
|  | Lyso-Sensor Green | ↓↓↓ | - | Fig. 3E-F |
|  | AO staining | ↓↓ | - | Fig. 3G, Fig. S3D |
|  | RHCG expression (cells/tissues/IHC) | ↑↑/↑↑/↑↑ | - | Fig. 3I-J/P-Q, Fig. S3 F-H |
|  | JC-1 red/green | ↓↓ | - | Fig. 6B-C |
|  | ROS | ↑↑ | - | Fig. 6D |
|  | P62/LC3 expression (cells/tissues/IHC) | ↑↑/↑↑↑/↑↑/↑↑↑ | - | Fig. 7C-E/H/J |
|  | Number of autolysosomes per cell | ↑↑↑ | - | Fig. 7F/J |
| + 4-PBA (Ammonia Scavenger) | Ammonia levels | - | ↓↓ | Fig. 1J |
|  | Cell death / Renal injury | - | ↓ | Fig. 1I |
|  | Lysosomal/Mitochondrial ammonia | - | ↓/↓↓ | Fig. 4A, Fig. 6F |
|  | Cath-D activity | - | ↑↑ | Fig S4 A |
|  | V-ATPase B2 expression (cells/tissues) | - | ↑/↑ | Fig S4 B-E |
|  | Lyso-Tracker Red | - | ↑ | Fig. 4B |
|  | Lyso-Sensor Green | - | ↑↑ | Fig. 4C |
|  | AO staining | - | ↑ | Fig. 4D-E |
|  | RHCG expression (cells/tissues/IHC) | - | ↑/↑/↑/↑↑ | Fig. 4F-K |
|  | JC-1 red/green | - | ↑ | Fig. 6G |
|  | ROS | - | ↓ | Fig. 6H-I |
| + CB-839 (GLS1 Inhibitor) | Ammonia levels | - | ↓↓ | Fig. 2E |
|  | Cell death / Renal injury | - | ↓/↓↓ | Fig. 2F-G/L-M |
|  | Serum UA/BUN/CRE/XOD | - | ↓↓/↓↓/↓/↓↓ | Fig. S2 B-E |
|  | GLS1 expression (cells/tissues/IHC) | - | ↓/↓/↓↓ | Fig. 2D/I/J-K |
|  | Lysosomal ammonia | - | ↓ | Fig. 5A |
|  | Cath-D activity | - | ↑↑ | Fig S4 B-F |
|  | V-ATPase B2 expression (cells/tissues) | - | ↑/↑↑ | Fig S4 G-J |
|  | Lyso-Tracker Red | - | ↑ | Fig. 5B |
|  | Lyso-Sensor Green | - | ↑↑ | Fig. 5C |
|  | AO staining | - | ↑ | Fig. 5D-E |
|  | RHCG expression (cells/tissues/IHC) | - | ↓↓/↓↓/↓/↓ | Fig. 5F-K |
| RHCG Overexpression | Lysosomal/ Mitochondrial ammonia | ↑/↓ | - | Fig. 3K |
|  | Cell death / Renal injury | ↑↑↑ | - | Fig. 3L, Fig. S3J |
|  | Cath-D activity | ↓↓ | - | Fig. S3I |
|  | V-ATPase B2 expression (cells/tissues) | ↓↓ | - | Fig. S3M |
|  | Lyso-Tracker Red | ↓↓↓ | - | Fig. 3M, Fig. S3K |
|  | Lyso-Sensor Green | ↓↓↓ | - | Fig. 3N |
|  | AO staining | ↓↓ | - | Fig. 3O, Fig. S3L |
| + 3-MA (Early Autophagy Inhibitor) | P62/LC3 expression (cells/tissues/IHC) | - | ↓/↓/↓/↓↓ | Fig. 8A-B/M-I, Fig. S5A/C |
|  | Number of autolysosomes per cell | - | ↓↓ | Fig. 8D |
| + Baf A1 (Late Autophagy Inhibitor) | P62/LC3 expression (cells/tissues/IHC) | - | ns | Fig. 8E-F/M/J, Fig. S5B-C |
|  | Number of autolysosomes per cell | - | ns | Fig. 8H |
| NH_4_CL | Cell death | ↑↑ | - | Fig. 1F |

↑ increase, ↓ decrease (number of arrows indicates significance)
